# Factors Influencing Safety on Construction Projects (fSCPs): Types and Categories

**DOI:** 10.3390/ijerph182010884

**Published:** 2021-10-16

**Authors:** Felipe Muñoz-La Rivera, Javier Mora-Serrano, Eugenio Oñate

**Affiliations:** 1 International Centre for Numerical Methods in Engineering (CIMNE), C/Gran Capitán S/N UPC Campus Nord, Edifici C1, 08034 Barcelona, Spain; mora@cimne.upc.edu (J.M.-S.); onate@cimne.upc.edu (E.O.); 2 School of Civil Engineering, Universitat Politècnica de Catalunya, Carrer de Jordi Girona, 1, 08034 Barcelona, Spain; 3 School of Civil Engineering, Pontificia Universidad Católica de Valparaíso, Av. Brasil 2147, Valparaíso 2340000, Chile

**Keywords:** safety on construction projects, safety performance, construction, accident

## Abstract

Due to the fact of activity, environment and work dynamics, the construction industry is characterised by high accident rates. Different initiatives have emerged to reduce these figures, which focus on using new methodologies and technologies for safety management. Therefore, it is essential to know the key factors and their influence on safety in construction projects (fSCPs) to focus efforts on these elements. Through a systematic literature review, based on PRISMA methodology, this article identifies, describes and categorises 100 factors that affect construction safety. It thus contributes by providing a comprehensive general framework, unifying previous studies focused on specific geographic areas or case studies with factors not considered or insufficiently disaggregated, along with an absence of classifications focused on understanding where and how factors affect the different dimensions of construction projects. The 100 factors identified are described and categorised according to the dimensions and aspects of the project in which these have an impact, along with identifying whether they are shaping or immediate factors or originating influences for the generation of accidents. These factors, their description and classification are a key contribution to improving the systematic creation of safety and generating training and awareness materials to fully develop a safety culture in organisations.

## 1. Introduction

### 1.1. Construction Safety

The construction industry is relevant for national economic and social development. It represents 6% of the world’s gross domestic product (GDP) and is expected to reach more than 14% by 2030 [1]. In Europe alone, the industry provides 18 million jobs [2]. Unfortunately, however, the construction sector is one of the most dangerous industries globally, with the highest accident rates [3]. Compared with the manufacturing industry, the probability of suffering a serious accident in construction is 2.5 times higher, and the probability of a deadly accident is five times higher [4]. In global terms, between 30–40% of construction sector accidents are fatal [5]. In recent years, accident figures have varied little or have even increased and, thus, the sector’s concern regarding these issues has increased, giving rise to initiatives towards a zero accident vision [6] focused on the systematic and profound improvement of safety in construction [7,8].

High accident rates in construction represent important social damage. Safety problems at the construction site affect the integrity of workers and their families, generating repercussions from the perspective of the social welfare of the sector with its employees and its social sustainability in general [9,10,11]. In addition, an accident affects the performance of the rest of the workers and work teams, making it necessary to reorganise work and provide psychological and emotional support [12,13]. Accidents also have repercussions on project productivity [14]. They lead to cost problems and delays in planning due to the interruption of work and its suspension for expertise. In addition, costs associated with workers’ compensation and civil liability costs represent considerable increases in budgets [15].

The construction industry is complex and dynamic; each project is unique, different professionals must interact (from different disciplines and companies) with different visions and products and must fit together to achieve the specific project aims [16,17]. As a consequence, the construction site is also peculiar and has characteristics that make safety control complex [18]. The construction site is subject to inclement weather, having little control over variables such as rain, wind or light levels [19]. The presence of heavy machinery and dangerous tools, the transport and use of materials on a large scale and the diversity of workgroups, roles and levels of workers’ training generates a heterogeneous work environment that is difficult to fully control [20]. Safety, therefore, is conditioned to different aspects, changing over time and with diverse responsible agents [21].

With a view to improving the future of construction, health and safety management in construction is key. This includes a series of activities focused on designing, monitoring and controlling occupational hazards in the sector, their mitigation and protection measures. The objective is to achieve the safe execution of work, focusing on eradicating accidents (goal: zero accidents) [22]. However, despite efforts in the sector, the construction industry worldwide is still characterised by high indicators of occupational accidents [23]. Most of these are attributed to the lack of proactive and preventive plans and measures such as risk identification and control, worker training and safety awareness and education. In addition, the lack of knowledge management of construction accidents does not allow for efficient feedback and learning [24]. Therefore, it is relevant to improve safety management to identify and understand which factors influence safety performance in construction in order to focus efforts on these aspects [25].

### 1.2. Factors Influencing Construction Safety

Construction safety performance is associated with different factors already studied by many different authors. From the perspective of safety plan management, Aksorn and Hadikusumo [26] identified four dimensions that these plans should consider: worker involvement, safety prevention and control system, safety arrangement and management commitment. Of these, Bavafa et al. [27] indicated that those aspects related to the safety responsibilities of each worker, personnel selection and subcontracts and employee involvement and safety evaluation are critical elements in safety programmes. Thus, organisational management is identified as a critical factor in safety management [28]. This allows the company’s dynamics and projects to be better understood and for the broad promotion of a safety-oriented organisational culture [29]. Thus, together with efficient information and material management and the use of technological tools, it is possible to develop a resilient safety culture in construction projects [30,31,32].

Several other authors have described lists of factors that affect construction safety according to studies carried out in different countries: Winge et al. [33] in Norway, Memon et al. [34] in Pakistan, Chen et al. [35] in China, Low et al. [36] in Hong Kong, Usukhbayar and Choi [15] in Mongolia, Yap and Lee [10] in Malaysia, Nadia et al. [5] in the United States and Chen et al. [35] in Taiwan. The factors associated with the worker and the work team include the company’s internal organisation and management, safety regulations, workplace conditions, supervisory aspects, worker training and individual responsibilities. Mohammadi et al. [37] identified 90 factors and categorised them into 13 groups, describing potential interactions between these categories. Winge et al. [7] identified 23 factors, grouped into four categories, which they placed and related in a construction accident causation framework and provided approximations regarding the level of influence (shaping factors, immediate factors or original influences) for the generation of accidents.

### 1.3. Research Objectives

There are previous studies on factors affecting construction safety, mainly focused on local studies or industries in specific countries validated through surveys and interviews with professionals and companies in each study region. On the other hand, there is evidence of global research that classifies and categorises relevant issues but is limited to a small number of factors. Other studies consider many factors but fail to group and identify their typologies of action. This research aimed to fill the identified research gaps by developing a comprehensive framework proposal that details the factors that influence safety in construction, classifies them and identifies their roles in the prevention of accidents. Thus, the objectives of this work were to:

1. Identify and describe the factors that influence safety in construction;
2. Categorise and group the factors according to the different dimensions and aspects of the project;
3. Classify factors according to whether these are shaping factors, immediate factors or original influences for the generation of accidents and frame them in a Construction Accident Causation Framework.

## 2. Research Methodology

**Figure 1:**
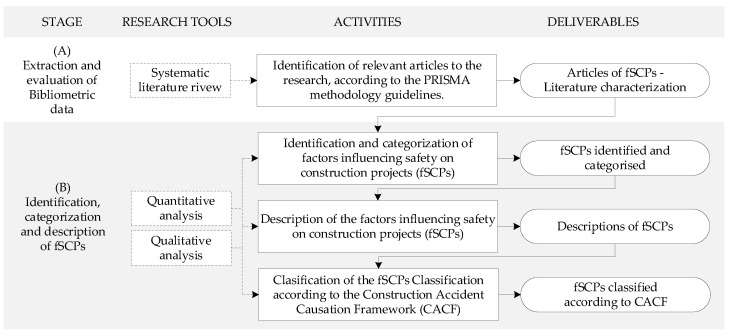
General research methodology.

This research identified the factors influencing safety on construction projects (fSCPs) to provide a comprehensive general framework for researchers in construction health and safety to focus their efforts for continuous improvement, especially considering the increase in the development and application of new technologies and methodologies in this area of study. The research was based on a systematic review, a quantitative analysis of the data obtained and qualitative analyses for the definition, classification and description of the factors identified. The research methodology was divided into two stages: (A) extraction and evaluation of bibliometric data based on PRISMA methodology; (B) identification, categorisation and description of fSCPs based on quantitative and qualitative analyses. Figure 1 shows the methodology workflow.

### 2.1. Extraction and Evaluation of Bibliometric Data

**Figure 2:**
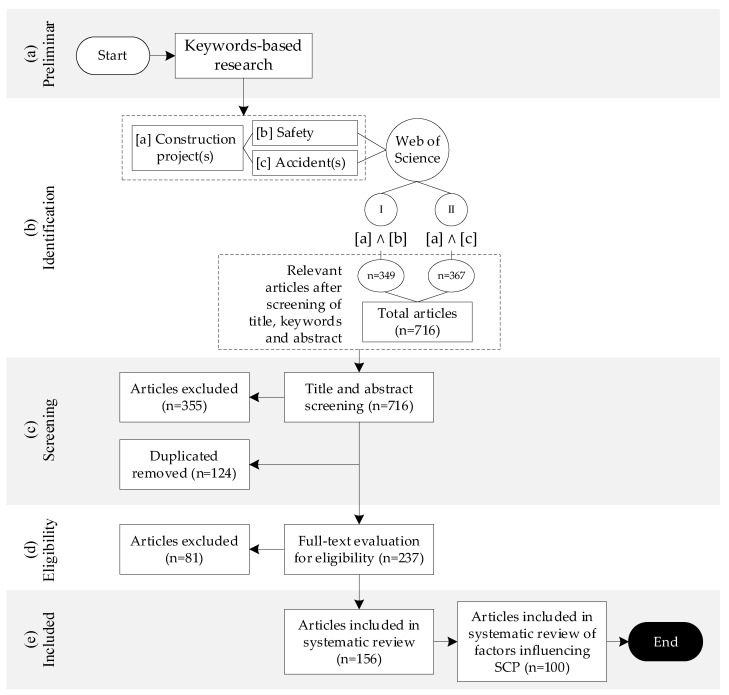
The PRISMA flow diagram for this systematic literature review.

For the identification, search, selection and evaluation of studies, the PRISMA (Preferred Reporting Items for Systematic Reviews and Meta-Analyses) methodology was used [38,39]. The PRISMA methodology allows for the search and selection of scientific articles for a systematic literature review. The methodology consists of five stages: (a) preliminary, (b) identification, (c) screening, (d) eligibility and (e) included, which range from defining the criteria for the search topics to the selection of the articles to be considered for the study. The iterative characteristic of the process proposed by PRISMA makes it possible to approach a wide variety of articles and, in turn, rigorously refine the selection, gathering of articles that are relevant to the research objectives and reduction of potential biases in the selection process. First, keywords and search databases are indicated. Then, the inclusion and exclusion criteria to filter the studies retrieved are indicated. This method was used to filter the studies retrieved. Figure 2 shows the PRISMA flow diagram for this systematic literature review.

#### 2.1.1. Selection of Database and Keywords

The Web of Science database was assessed to obtain the publications for this study due to the fact of its comprehensive coverage and its international prestige, both in general aspects and in the topics of this research [40]. The keywords used were: (a) “construction project(s)”; (b) “safety”; (c) “accident”. Thus, two searches were carried out, and two search combinations were used in each: (I) (a) “construction project(s)” ∧ (b) “safety”; and (II) (a) “construction project(s)” ∧ (c) “accident”.

#### 2.1.2. Inclusion and Exclusion Criteria

Inclusion and exclusion criteria were defined to filter the retrieved studies and retain only the relevant publications. The inclusion criteria included: (1) studies focusing on factors affecting construction safety; (2) studies addressing causes of construction accidents; (3) studies addressing (safety) risks in construction projects; (4) studies published in peer-reviewed journals and conference proceedings. The exclusion criteria were: (1) studies focusing on the use or development of specific construction safety technologies and not on fSCPs; (2) studies showing specific case studies (analysis of only one construction site, for example); (3) studies published in languages other than the English language; (4) studies without the full text available.

#### 2.1.3. Screening and Evaluation of Retrieved Studies

As of March 2021, 716 articles were retrieved from the Web of Science database: 349 articles from search combination I and 367 articles from search combination II. According to Figure 2, using the PRISMA methodology, titles, keywords and abstracts were reviewed for each of the 716 articles. Following this, 355 articles were excluded as they were considered irrelevant. In addition, 124 duplicate articles were removed. Next, 237 articles were reviewed in full, excluding 81 articles. Finally, 156 articles were considered relevant to the topics studied, and after applying filters according to the inclusion and exclusion criteria (see Section 2.1.2.), 100 were selected to identify factors influencing safety in construction projects. 

#### 2.1.4. Screening Article Coverage and Presence of Bias

While the PRISMA systematic review methodology is widely recognised and used, it may not guarantee complete coverage of all the papers that should be reviewed. Nevertheless, the approach used was sufficient to identify a considerable number of significant papers in the study area of interest, perform analyses upon them and draw conclusions. On the other hand, for the identification and inclusion of articles, a “selection bias” (error due to wrong sample or study group selection) could be considered in some studies, which could affect the results of such research (e.g., in country-specific studies) [41]. However, this aspect does not discredit a study; it only warrants critical analyses of the context and applicability of its results. With this, and considering the selection, inclusion and exclusion criteria established, the selected articles do not show bias that influences the focus and objectives of the research.

### 2.2. Identification, Categorisation and Description of fSCPs

Based on the background obtained through the systematic literature review, this research aimed to (1) identify and describe the factors that influence safety in construction, (2) categorise and group the different factors according to the different dimensions and aspects of a construction project and then (3) classify them according to whether they are shaping factors, immediate factors or original influences for the generation of accidents, framing them in a Construction Accident Causation Framework. 

## 3. Literature Characterisation

Based on the literature review, in this section, the results of the extraction and evaluation of bibliometric data are shown. This includes the annual trend in published articles, journals where the articles were published and identification of research focus trends.

**Figure 3:**
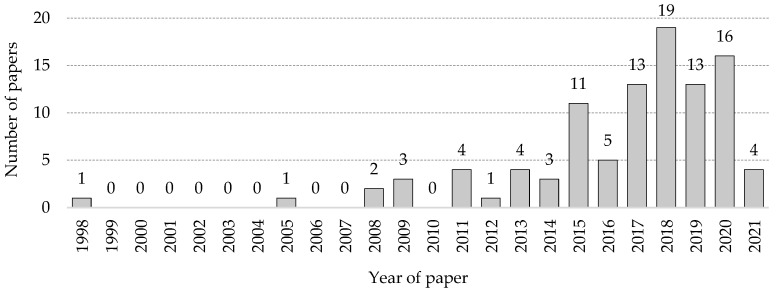
Annual distribution of papers.

Figure 3 shows the annual distribution of articles selected in this research, according to the guidelines in Section 2. One hundred articles were selected between 1998 and 2021. Before 2011, only seven articles were found. At first glance, it was evident that the number of articles had increased since 2015 (in the year 2021, given the date of conducting this research, articles were retrieved only up to March). The growing concern in the industry for its high accident rates, together with the digitisation of the sector in recent years (which has allowed an improvement in the capture and management of construction data), could be responsible for the increase in the number of these types of studies. It is important to remember that the Construction (Health, Safety and Welfare) Regulations came into force on 2 September 1996, with the National Occupational Research Agenda beginning to conduct research to mitigate the accident rate but apparently without a substantial number of publications, far from the current figure.

**Figure 4:**
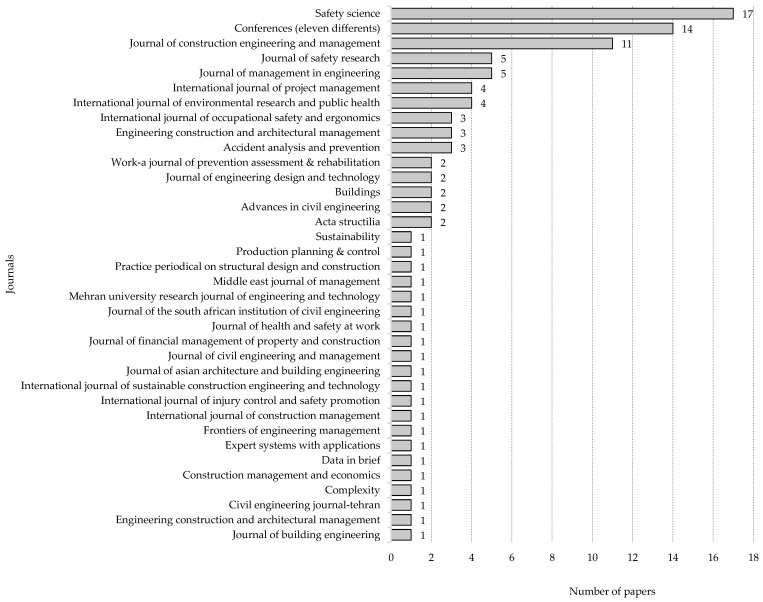
Numbers of papers published in the journals.

Figure 4 shows the journal titles and the number of articles found for each of them. Of the total number (100) of articles, 86% corresponded to journal articles and 16% to conference articles. The journals with the highest number of articles were Safety Science (17) and Journal of Construction Engineering and Management (11).

Based on these 100 papers selected, 100 factors influencing safety on construction projects (fSCPs) were identified and described, and categories were established to organise them. Each paper addressed various factors and aspects of interest, contributing quantitatively and qualitatively to fulfilling the research objectives. Table S1 in the Supplementary Materials contains the details of the intersection between the 100 factors identified and the 100 papers collected. However, 17 papers were identified as relevant because their results contributed more directly to the objectives of this research. The approaches to the search for factors and classifications presented in these investigations have served as input for constructing the proposal presented in this research. Some classifications names of factors and categories were considered and/or adapted for this research. Table 1 shows the title, year of publication, journal, field and a description of each study, highlighting the contributions and limitations.

## 4. Categorisation of the fSCPs

This section proposes a categorisation of the fSCPs by merging and filtering the different potential categories and classifications established by the authors from the previous literature review such as:

- Organisation, classifications associated with project and company management, security policies and the organisation’s culture with regard to security [15,33];
- Elements of the worksite are associated with the construction site’s characteristics and the conditions to avoid or facilitate accidents [28,34];
- The condition of work equipment, machinery and tools as an important factor that needs special monitoring considerations [7,20];
- The human factor, worker and the work team: attitude to risk-taking, risk perception, worker training and skills and attitudes to supervision and interaction with other workers [10,35,36].

On the other hand, categories of factors have been proposed from the perspective of resilient safety management, i.e., the generation of a capacity for “defence” before an accident and “recovery” after an accident. These consider dimensions associated with organisational management, personnel management associated with the individual safety responsibility of each employee, information management of safety and accident processes and the correct administration and maintenance of materials and equipment involved in safety [31]. In addition, safety awareness and organisational culture are considered relevant aspects [30]. 

Thus, four categories were established with the aim of grouping together the main dimensions to be considered when managing the factors that affect safety in construction:

- General aspects (A): the aspects that the company must consider at a global level to establish particular plans for its projects. It considers elements from the organisational point of view, regulations, financial aspects and organisational productivity, culture and climate towards safety which must permeate all organisational members, together with elements associated with previous lessons and accident rate metrics to establish safety management programmes and systems;
- Materials and equipment (B): the condition and characteristics of work materials and equipment about their influence on the potential for accidents. These are all the objects used in the workplace, focusing on the elements that physically can be dangerous such as heavy machinery and work tools;
- Construction site (C): the conditions of the construction site and how the characteristics of the workplace, in combination with work processes, can promote or prevent the occurrence of accidents. Each construction site is unique and different from the others, requiring a different review and safety measures. These are relevant aspects of safety management;
- Human aspects—worker and work team (D): These are aspects that influence human performance and, therefore, safety as well. The competencies and motivations of workers, from the perspective of individual responsibility for safety issues, together with the collective responsibility of the whole work team are included. Aspects of interaction and communication are considered key and relevant.

**Figure 5:**
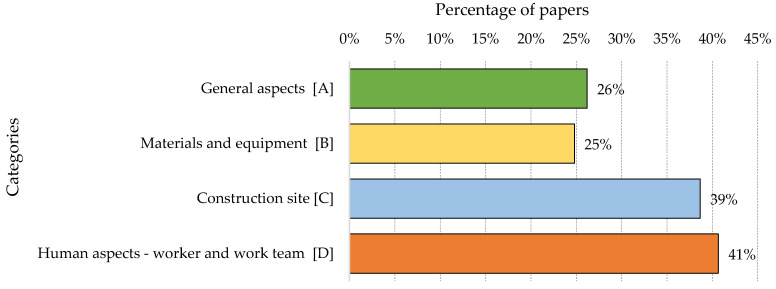
Percentage distribution of factors in each of the four categories.

Figure 5 shows the percentage distribution of factors in each of the four categories for the total amount of research reviewed. The presence of factors in the categories of “General aspects” and “Materials and equipment” was similar (26% and 25%, respectively). On the other hand, there was a higher presence of factors associated with “Construction site” (in 39% of the articles) and “Human aspects—worker and work team” (in 41% of the articles).

**Figure 6:**
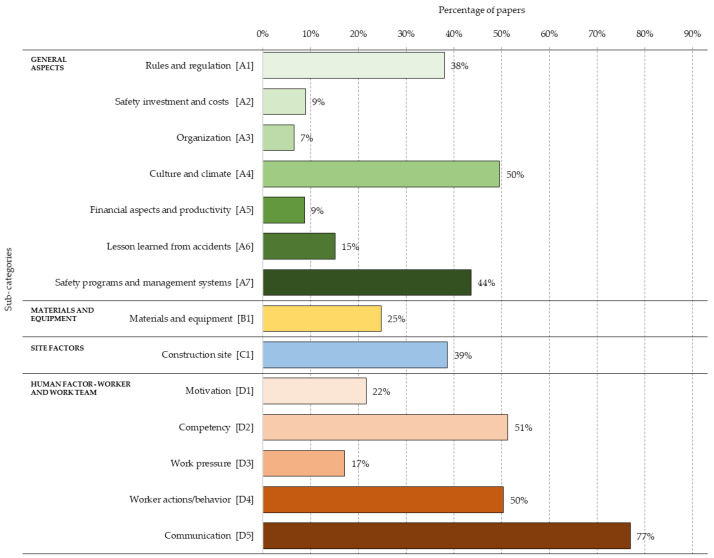
Percentage distribution of factors in each of the 14 sub-categories.

Before the global disaggregation of the 100 identified factors, sub-categories were established to allow an intermediate disaggregation of the four categories described above. These were generated by merging and filtering the different subcategories identified in the literature review. Figure 6 shows the 14 established sub-categories according to the four categories initially defined. For each sub-category, the percentage of papers considering factors from that category is shown. In “General aspects (A)”, seven sub-categories were considered: “Rules and regulation” (38%), “Safety programmes and management systems” (44%) and “Culture and climate” (50%) were the categories with the highest presence in the review. Sub-categories for “Materials and equipment (B)” and “Construction site (C)” were not considered, as they were already considered to be final classifications. For “Human aspects—worker and work team (D)”, five sub-categories were defined. Of these, “Communication” had the highest presence (in 77% of the papers), followed by “Competency” (51%) and “Worker actions/behaviour” (50%).

## 5. Descriptions of the fSCPs

### 5.1. General Aspects (A)

**Figure 7:**
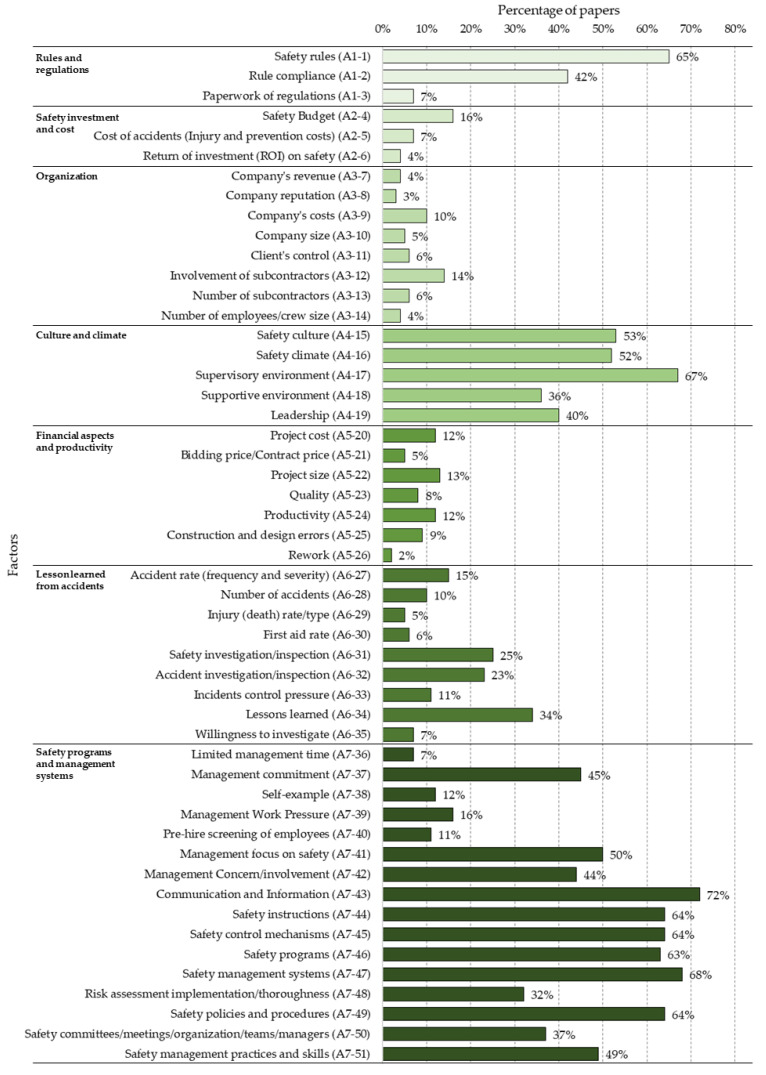
Percentage distribution of factors in the “General aspects (A)” category.

Each of the factors are described below according to the sub-category. Figure 7 shows the percentage presence of each of the 51 factors for the “General aspects” category.

#### 5.1.1. Rules and Regulation (A1)

Associated with regulatory aspects that influence safety. The following factors are considered:

- A1-1—Safety rules: general or specific company regulations on safety issues that must be applied to projects [7,26,34,35,43,44];
- A1-2—Rule compliance: level of compliance with the general or specific regulations of the company on security matters that must be applied to the projects [15,27,31,42,45,46];
- A1-3—Paperwork of regulations: compliance with the procedures on security issues that must be applied to the projects [15,27,47].

#### 5.1.2. Safety Investment and Cost (A2)

Related to economic aspects that could influence safety. The following factors are considered:

- A2-4—Safety budget: The actions that the company will be able to implement in the organisation. The size of the security budget may be limited to the economic conditions of the project but also to the level of importance that the company gives to this aspect [7,33,48,49,50,51,52];
- A2-5—Cost of accidents (injury and prevention costs): refers to the costs that the company must pay for accident prevention, accident treatment and associated compensation [15,53,54];
- A2-6—Return on investment (ROI) on safety: refers to the company’s vision regarding the benefits of investing in safety, not only for the expenses not incurred in avoided accidents but also in the organisational climate, the well-being of the workers and the reputation of the company [15,54].

#### 5.1.3. Organisation (A3)

Associated with general company indicators that could influence safety. The following factors are considered:

- **A3-7—Company’s revenue:** related to the difficulty in implementing and controlling security systems in projects [15,20,52,54];
- **A3-8—Company reputation:** accident indicators are directly related to the company’s reputation, thus concern for the company’s reputation could be an essential factor in promoting concern for safety [15,49,54];
- **A3-9—Company’s costs:** linked to the difficulty in implementing and controlling security systems in projects [20,33,55];
- **A3-10—Company size:** it affects the complexity of implementing and controlling security systems in projects [20,42,49];
- **A3-11—Client’s control:** the client’s direct influence on the company’s security measures should improve associated management processes [15,33,49,54,56];
- **A3-12—Involvement of subcontractors:** alignment of safety culture among the organisations working together is key to implementing and controlling security systems in projects [7,10,51,56,57];
- **A3-13—Number of subcontractors:** same as previous, this factor influences the difficulty in implementing and controlling security systems in projects [7,15,54,58];
- **A3-14—Number of employees/crew size:** the greater the number, the greater the difficulty in implementing and controlling security systems in projects [7,15,54,59].

#### 5.1.4. Culture and Climate (A4)

This is related to the aspects of perception and awareness of security in the organisation that provide a safe environment. The following factors are considered:

- **A4-15—Safety culture:** It is the way things are done in the company, related to actions, practices, ways of working, among other aspects, which lead to the identification of a series of common elements promoting safety in the organisation [5,15,35,43,44,60];
- **A4-16—Safety climate:** the feeling that the workers of a company have regarding safety. This is reflected in knowing the actions, protocols and measures implemented, making workers feel safe (or unsafe) [28,29,30,47,60];
- **A4-17—Supervisory environment:** refers to the existence of different instruments for the supervision of safety in the company [10,34,61,62,63,64];
- **A4-18—Supportive environment:** the work environment is adequate and promotes safety. It refers not as much as the physical environment but to the systems and protocols that the company has implemented to promote security [5,65,66,67,68];
- **A4-19—Leadership:** associated with the company’s ability to lead transformation processes on safety issues and influence workers to promote responsibility in these aspects [15,30,34,69,70,71].

#### 5.1.5. Financial Aspects and Productivity (A5)

These aspects are focused on how productivity (mainly time, cost and quality) could influence the safety performance of projects. The following factors are considered:

- **A5-20—Project cost:** directly related to the budget slack applied for safety and the level of importance that the company places on safety [5,33,53];
- **A5-21—Bidding price/contract price:** same as previous, this factor affects the budget slack for safety and priority of safety for the company [7,15,49];
- **A5-22—Project size:** it impacts the budget slack for safety and the level of importance that the company places on safety [33,42,72,73];
- **A5-23—Quality:** this factor affects the project’s quality requirements, which leads to an increase or decrease in the importance of safety in the tasks performed [7,15,74];
- **A5-24—Productivity:** productivity increases could overstress workers (mainly in time) and lead to the generation of accidents [15,75,76,77];
- **A5-25—Construction and design errors:** these factors can impact the performance of workers, generating incorrect actions in the workplace [7,15,53,78,79];
- **A5-26—Rework:** generates an increase in project costs and times, which influences safety [7,15].

#### 5.1.6. Lesson Learned from Accidents (A6)

It refers to the follow-up, measurement, monitoring and analysis of the trends of the indicators associated with accident management and potential actions to mitigate them. Factors considered:

- **A6-27—Accident rate (frequency and severity):** see [31,61,80,81];
- **A6-28—Number of accidents:** see [15,31,82,83];
- **A6-29—Injury (death) rate/type:** see [15,31,84];
- **A6-30—First aid rate:** see [15,31];
- **A6-31—Safety investigation/inspection:** there are regular investigations and inspections of security systems [30,42,85,86];
- **A6-32—Accident investigation/inspection:** the causes of accidents are investigated, and there is concern about their identification [31,42,80];
- **A6-33—Incidents control pressure:** there is an excessive tendency to control incidents which causes pressure on workers, generating adverse effects on safety issues [31,61,69];
- **A6-34—Lessons learned:** the company uses information from subsequent accidents for continuous learning and improvement of its safety plans and systems [5,30,43,74,87,88];
- **A6-35—Willingness to investigate:** the company’s tendency to exhaustively investigate the causes of accidents [15,31,49].

#### 5.1.7. Safety Programmes and Management Systems (A7)

Plans, programmes and the company’s dedication to putting safety measures in their projects. Focus is on the company culture. The following factors are considered:

- **A7-36—Limited management time:** A reduced time that the organisation spends on security management will be reflected in inconsequential actions on these issues. This creates a perspective that these aspects are of little importance for the workers [7,33,53];
- **A7-37—Management commitment:** management’s involvement with safety in the organisation influences each worker’s commitment to these aspects [26,28,35,57,68];
- **A7-38—Self-example:** it serves as examples for workers to join these initiatives on safety issues associated with the actions that the company carries out and the measures it establishes [7,30,43,73,89];
- **A7-39—Management work pressure:** the pressure that management puts on workers, such as times, forms and work objectives, contributes to generating much riskier scenarios [7,31,62,90];
- **A7-40—Pre-hire screening of employees:** consideration of the capabilities and safety training that workers have at the time of hiring [7,56,61,83,91];
- **A7-41—Management focus on safety:** The approaches in which the company’s management manages safety. It can be reactive, proactive, rigorous and light approaches, among others. The approach is reflected in the safety management systems implemented [10,29,42,47,67];
- **A7-42—Management concern/involvement:** a company’s management must be committed to establishing safety management protocols and systems, reflected in concrete actions at the company [29,35,36,57];
- **A7-43—Communication and information:** The company defines processes for the communication of security protocols. The delivery of information is timely and understandable to all workers [33,34,36,42,92,93];
- **A7-44—Safety instructions:** the company defines instructions and manuals for the safe execution of tasks and protocols to be followed in each project [15,26,33,42,85];
- **A7-45—Safety control mechanisms:** the company defines tools for safety control in the projects [15,34,36,46];
- **A7-46—Safety programmes:** the company defines policies, strategies and tools to promote safety in its projects [7,15,20,26,45];
- **A7-47—Safety management systems:** the company has safety management systems to implement and control safety in its projects [5,15,28,47,94];
- **A7-48—Risk assessment implementation/thoroughness:** the company assesses the risks of its projects using defined protocols [15,42,62,95];
- **A7-49—Safety policies and procedures:** the company has internal regulations on safety issues which it implements in its projects [26,34,36,69,96];
- **A7-50—Safety committees/meetings/organisation/teams/managers:** the company has established different instances and teams concerned with safety in the organisation [5,35,54,97,98];
- **A7-51—Safety management practices and skills:** the company has developed capabilities and good practices in safety issues and implements them in the projects it develops [15,26,34,50,99,100].

### 5.2. Materials and Equipment (B)

**Figure 8:**
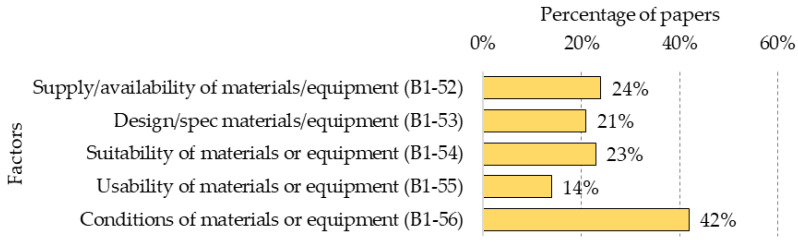
Percentage distribution of factors in the “Materials and equipment (B)” category.

Materials and equipment correspond to all the objects used in the workplace with special attention to those elements that can be physically dangerous, particularly heavy machinery. Figure 8 shows the percentage presence of each of the five factors for this category, listed below.

- **B1-52—Supply/availability of materials/equipment:** lack of required materials and equipment, which may lead to the use of non-appropriate alternatives that could create unsafe working conditions [10,15,48,63,64,79,100];
- **B1-53—Design/specification materials/equipment:** poor technical specifications and/or design of materials or equipment for the tasks to be performed, especially in terms of usability and safety, generating risky actions [15,31,90,101,102];
- **B1-54—Suitability of materials or equipment:** Use of materials or equipment with functionalities that are not specific for the task to be performed. Using them in inappropriate actions could generate unsafe working conditions [10,36,43,57,103];
- **B1-55—Usability of materials or equipment:** functionality problems of materials or equipment that perturb their regular use, generating unsafe working conditions [30,35,64];
- **B1-56—Conditions of materials or equipment:** poor condition of materials or equipment that could affect the performance of tasks and lead to unsafe working conditions [10,26,28,55,73].

### 5.3. Construction Site (C)

**Figure 9:**
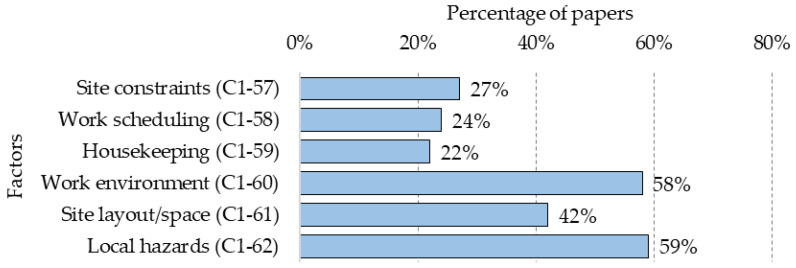
Percentage distribution of factors in the “Construction site (C)” category.

Worksite characteristics are key aspects for any safety plan, taking also into account that each building site is unique and different from the others, requiring a specific review and safety measures. Figure 9 shows the percentage presence of each of the following six factors for this category:

- **C1-57—Site constraints:** The limitations of the workplace require tasks to be performed in a particular way and only for that place. The use of machinery, tools and the performance of tasks at the construction sites are key aspects to assess the related hazards. Work processes and safety measures must be adapted to the constraints of the work site [33,34,78,80,103];
- **C1-58—Work scheduling:** work dynamics affect the health and safety of workers and are associated with the planning of the actions to be carried out in terms of time and distribution [5,33,52,58,100,104,105];
- **C1-59—Housekeeping:** orderly/disorderly conditions of the worksite may disturb the work process and generate risks, be it of materials, equipment, machinery, waste management, etc. [10,42,73,82];
- **C1-60—Work environment:** environmental conditions (physical or climatic) influence the occurrence of accidents, such as wet, noise, high/low lighting, thermal stress, etc [5,26,28,99];
- **C1-61—Site layout/space:** the level by which the workplace configuration contributes to generating accidents and increasing the levels of risk during the execution of the work [26,28,33,46];
- **C1-62—Local hazards:** specific construction site hazards and risks that have to be avoided or mitigated through safety management and planning before workers start working at the site [5,20,34,35,106,107].

### 5.4. Human Aspects—Worker and Work Team (D)

**Figure 10:**
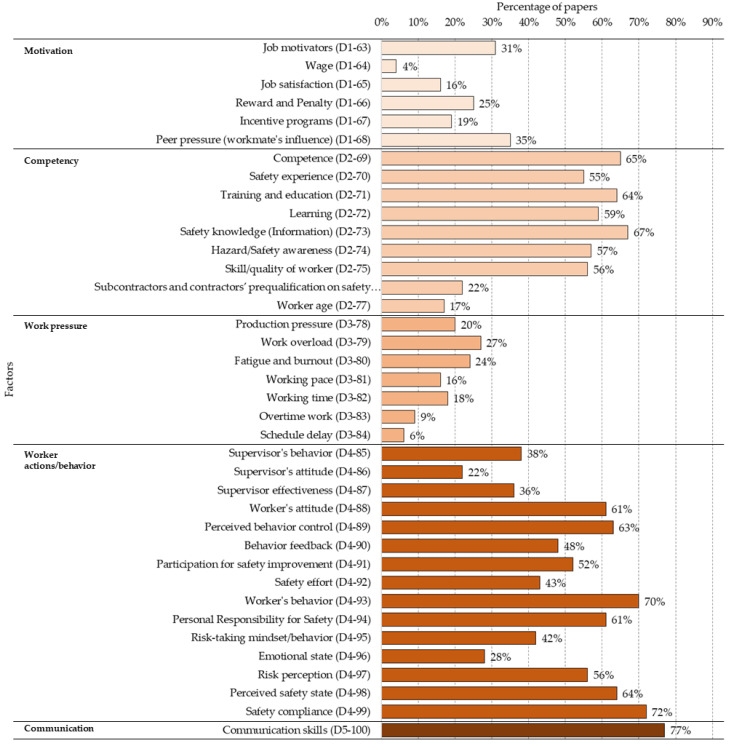
Percentage distribution of factors in the “Human aspects—worker and work team (D)” category.

Human aspects refer to any aspect that influences human performance and, therefore, safety as well including workforce capabilities, motivation, human constraints, the perception of workers about the work environment, emotions, skills evolution and skills themselves, training and awareness, etc. Figure 10 shows the percentage presence of each of the 38 factors for this category.

#### 5.4.1. Motivation (D1)

Those elements that promote more or less satisfactory working conditions, attitude and motivation of the worker to perform his tasks.

- **D1-63—Job motivators:** the employee’s motivation to perform their work and their willingness to perform (better or worse) the assigned tasks [5,15,29,59,99,108,109];
- **D1-64—Wage:** the worker’s level of satisfaction with their salary [5,15,49];
- **D1-65—Job satisfaction:** how comfortable they feel with the assigned work [30,33,52,74,76];
- **D1-66—Reward and Penalty:** relationship between the performance of their tasks with their feeling of being comfortable with the work they are doing [42,43,61,105,110];
- **D1-67—Incentive programmes to motivate workers:** attention to safety issues when linked to potential rewards for good (and safety) performance [5,28,110,111];
- **D1-68—Peer pressure (workmate’s influence):** Influence of co-workers in terms of direct pressure or, indirectly, behaviour on the safety performance of individual workers. This “mirror” behaviour can come from positive or negative examples (it is not uncommon for some teasing from co-workers for following protocols, use of protective elements, among others) [15,26,96,112,113,114].

#### 5.4.2. Competency (D2)

This factor includes any capabilities and characteristics that the worker and the work team possess, leading to a safer work environment.

- **D2-69—Competence:** skills that the worker has developed in safety matters that make him/her have safer behaviours and awareness of the risks associated with the tasks performed [10,26,29,99,115];
- **D2-70—Safety experience:** ability of the worker to securely face tasks due to the fact of previous training or experiences [20,28,35,82,102,116];
- **D2-71—Training and education:** Courses and learning that the worker has taken on safety issues. It is important to distinguish between general and specific training, depending on the work to be performed. It is clear that learning about ergonomics in the office is not particularly helpful if you must operate a drilling machine [7,31,35,37,102];
- **D2-72—Learning:** Knowledge and skills that the worker possesses on safety issues. It is essential to identify how or by what methods the worker acquires these skills and their significance [7,10,31,35,37];
- **D2-73—Safety knowledge (Information):** The knowledge that the worker has on safety issues whether it be regulations, best practices or lessons learned, along with the level of access to this information. This is key to generating a long-term prevention culture [7,31,35,37,85];
- **D2-74—Hazard/Safety awareness:** Level of assimilation of the hazards and risks associated with the different tasks he/she performs, along with the consequences of his/her actions. Promoting more awareness about the consequences of safety (best or worse) practices makes behaviours much safer [20,34,106,117];
- **D2-75—Skill/quality of worker:** Level of professionalisation of the worker, both in the specific tasks performed and in safety knowledge. It is not necessarily just senior workers that have safer behaviours than junior ones, but there is a direct correlation with the qualifications of the workers [20,31,33,43,75];
- **D2-76—Subcontractors and contractors’ prequalification on safety:** evaluation of the safety knowledge and rules of subcontracted work teams and alignment with the rules of the principal contractor before they enter the worksite [15,34,53,83,117];
- **D2-77—Worker age:** related to much more experience in security issues at a practical level but also to the persistence of some bad practices. This is an important factor when identifying forms of training and developing an organisational safety culture [10,15,86,112,115,116].

#### 5.4.3. Work Pressure (D3)

Working conditions in terms of timing and performance of tasks, leading to unsafe actions.

- **D3-78—Production pressure:** it has direct repercussions on incorrect performances of tasks with an increase of the risks associated with their poor execution due to the time pressures and work methods [10,42,84,90,118];
- **D3-79—Work overload:** quite similar to the previous factor, it results in the incorrect performance of tasks and generation of risks associated with that poor execution [31,36,66,77,84];
- **D3-80—Fatigue and burnout:** leading to errors in the performance of tasks, along with a decrease in the worker’s attention to potential hazards, increasing the possibility of accidents [36,52,74,117,118];
- **D3-81—Working pace:** depending on the complexity of the tasks, an accelerated pace of work can lead to accidents [5,15,78,99];
- **D3-82—Working time:** depending on the complexity of the tasks, excessive work times could generate wear and tear on workers, leading to accidents [33,42,46,86];
- **D3-83—Overtime work:** could generate excessive physical wear and tear on workers, increasing the risks [31,42,55,84];
- **D3-84: Schedule delay:** Delays in work schedules or specific tasks can increase risks due to the pressure to increase the speed at which tasks are completed. For this reason, there are risks that would not exist without these deadlines [7,31,52,74].

#### 5.4.4. Worker Actions/Behaviour (D4)

These factors correspond to the characteristics of the workers, their actions and behaviours, together with the supervisory environment, which either promote or prevent unsafe actions.

- **D4-85—Supervisor’s behaviour:** supervisor’s direct actions to promote safe actions in the workplace [5,15,28,65,70];
- **D4-86—Supervisor’s attitude:** Ways in which the safety supervisor indicates standards, right and wrong actions, and gaps for improvement to workers. It is associated with the application of people management methods to create a safety culture and raise awareness and correct workers through dialogue, understanding and the use of social skills [10,28,36,89,119];
- **D4-87—Supervisor effectiveness:** results of the actions and methods performed by the supervisor to promote safe routines in the workplace [10,26,75,87,94];
- **D4-88—Worker’s attitude:** Corresponds to the worker’s willingness to learn and apply safety regulations and respect work protocols. It is also associated with the worker’s attitude when carrying out tasks that entail personal or collective risks and the worker’s decisions in these cases [20,26,97,120];
- **D4-89—Perceived behaviour control:** worker perception of control and monitoring of their actions at the workplace about safety issues, along with overall monitoring of safety at the site [5,15,20,72,113];
- **D4-90—Behaviour feedback:** the workers receives feedback on their safety behaviour, which allows for continuous improvement of their behaviour at the workplace [5,15,57,81,88,119];
- **D4-91—Participation for safety improvement (workers’ involvement and cognitive and emotional engagement):** The engagement of workers in the improvement of workplace safety, including their participation in training activities, but mainly referring to their attitudes and general promotion of safety [5,29,85,107,120];
- **D4-92—Safety effort:** The constant willingness of the worker to learn and comply with safety rules, along with promoting safe actions in the workplace. There is a constant concern for personal and collective safety [10,15,29,106,115];
- **D4-93—Worker’s behaviour:** Actions taken by the worker with direct causality to the occurrence of an accident (by their own or due to the actions by other workers). Includes unsafe actions, reactions, preventive routines, errors and incorrect execution of procedures [26,34,35,65];
- **D4-94—Personal Responsibility for Safety:** The individual workers’ awareness of their own safety, the consequences of their actions and limitations associated with risk levels and safe conditions. It considers a personal safety mindset as well as the safety consequences for co-workers [15,31,35,75];
- **D4-95—Risk-taking mindset/behaviour:** the performance of risky actions in the workplace because the worker assumes these as challenges or prioritises the performance of a task over the risk of performing it in a certain way [5,37,42,55,108,121];
- **D4-96—Emotional state:** a worker’s personal problems and situations can influence the poor execution of actions and lead to accidents [10,15,27,93,104,113];
- **D4-97—Risk perception:** a worker’s ability or judgement about the conditions of the workplace and the levels of risks they face. Although it is subjective, safety conditions and training are key to the correct perception of risks [7,27,28,45,60];
- **D4-98—Perceived safety state:** Corresponds to the worker’s perception of the safety of the workplace and the actions within it. If the safety conditions are correct, the worker feels that the workplace is safe, encouraging correct behaviour and promoting compliance with safety regulations [7,20,33,106,122,123,124,125];
- **D4-99—Safety compliance:** The worker complies with the safety rules and instructions for the correct execution of the tasks. The worker knows the safety regulations and applies them correctly [20,31,37,43,122,124,126].

#### 5.4.5. Communication (D5)

Communication refers to the messages, channels and protocols when an accident occurs, in the short term, but also to the creation of a culture of safety with a long-term vision.

- **D5-100—Communication skills:** Each worker and the work team’s ability to communicate correctly and transfer safety knowledge and the different instructions for the development of the activities. It also considers language limitations (spoken and written) [5,20,26,35,37,92].

## 6. Classification According to the Construction Accident Causation Framework (CACF)

After this extensive definition of categories and sub-categories addressing the key dimensions of safety, a further classification was necessary to better understand the relationships between them, how to prioritise them and, therefore, at what level the factors affect the potential occurrence of an accident. Haslam et al. [127,128] proposed the Construction Accident Causation Framework, later adapted by Winge et al. [7], with three factors for the generation of accidents: immediate factors, shaping factors and originating influences:

- The **originating influences** refer to aspects associated with organisational characteristics and different aspects of management and regulation that make up the environment within which safety measures are (or not) favoured and not directly related to accidents in the short-term but are essential for explaining the long-term causes;
- At an intermediate level, **shaping factors** positively or negatively drive actions in the workplace, generating conditions conducive to safety or the generation of potential risk situations;
- **Immediate factors** are those elements that are directly related to the occurrence or prevention of an accident.

**Figure 11:**
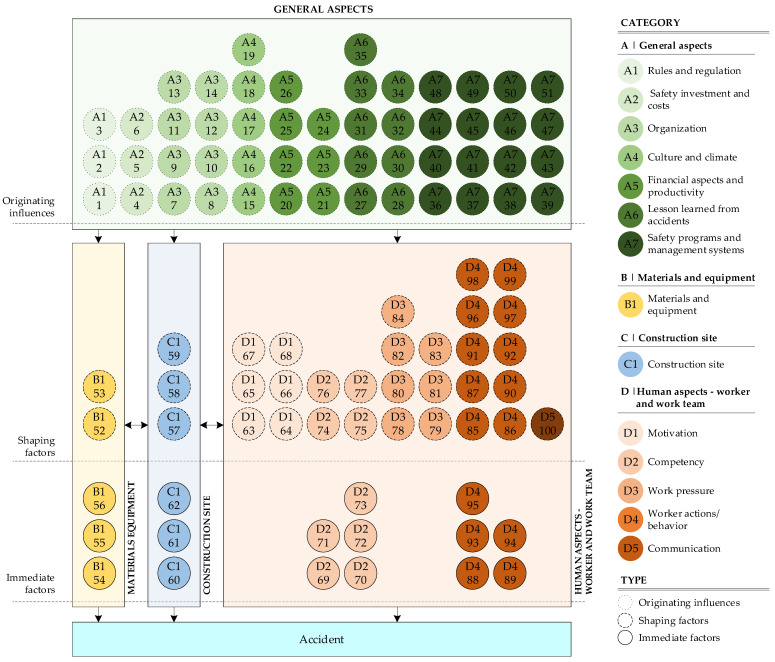
fSCPs mapped to the Construction Accident Causation Framework.

With this information and after analysing the reviewed papers, the 100 fSCPs were classified within the Construction Accident Causation Framework. According to this classification, Figure 11 shows the distribution of the various identified factors and whether these are immediate factors, shaping factors or originating influences. Therefore, the 51 factors in the “General aspects” category are classified as originating influences, since they constitute a general framework for security issues. The other three categories (“Materials and equipment”, “Construction site” and “Human aspects—worker and work team”) are distributed along shaping and immediate factors.

In Figure 11, it is possible to see that all the factors in the “General aspects” category are within originating influences, because these are elements that correspond to characteristics of the organisation and generate an environment for security (or insecurity). Thus, the subcategories’ factors associated with the organisation’s general characteristics (size, costs, etc.) and financial and productivity elements will give a general framework. With them, “Rules and regulations”, “Safety programs” and “Lessons learned” information will allow the implementation of better safety prevention measures for the worksite to foster a safety culture and climate in the organisation and all its projects.

In “Materials and equipment (B)”, the factors “Supply/availability of materials/equipment (B1-52)” and “Design/specifications of materials/equipment (B1-53)” are considered shaping factors because these are not directly related to the potential occurrence of an accident. Still, these may generate an unfavourable condition for the safe performance of a task in the workplace. For example, the unavailability of a material or a piece of equipment may cause the worker to substitute it for another with incorrect specifications, generating a potentially unsafe condition. On the other hand, the factors “Suitability of materials or equipment (B1-54)”, “Utility of materials or equipment (B1-55)” and “Conditions of materials or equipment (B1-56)” are immediate factors, because these are aspects that are directly related to the generation of a potential accident.

In “Construction site (C)”, “Site constraints (C1-57)”, “Work scheduling (C1-58)” and “Housekeeping (C1-59)” are considered shaping factors because the correct or incorrect management of these aspects could generate unfavourable conditions for safety. On the other hand, “Work environment (C1-60)”, “Site layout/space (C1-61)” and “Local hazards (C1-62)” are considered immediate factors because these could be directly related to a potential worker accident.

Finally, in “Human aspects—worker and work team (D)”, the factors in the subcategories of “Motivation”, “Work Pressure” and “Communication” are categorised as shaping factors, because these are elements that positively or negatively condition safety but are not directly related to the occurrence of accidents. On the other hand, it is possible to find shaping and immediate factors in the “Competency” and “Worker actions/behaviour” subcategories. Some elements generate potential unsafe conditions (for example, aspects associated with supervision, workers’ emotional state and perception, safety awareness, among others). In addition, other elements are directly related to a potential accident. Competencies and training, together with the worker’s responsibility and other factors, are essential.

**Figure 12:**
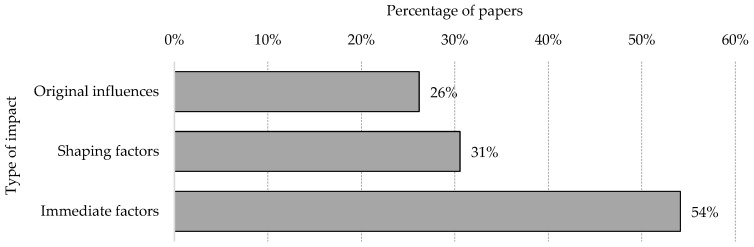
Presence of the factors in the review by types of impacts according to the CACF.

Figure 12 shows the presence of the factors in the review according to the types of impacts defined according to the Construction Accident Causation Framework (CACF). The immediate factors are present in 54% of the papers reviewed, followed by shaping factors (31%) and then originating influence factors (26%).

## 7. Discussion

### 7.1. Representative Use Cases

Construction sites are some of the most dangerous working places, and many efforts have been conducted to mitigate the high rate of accidents. There are some obvious dangers, such as working at heights or the use of the traditional heavy equipment or machinery, but there are others that are not so obvious or even hidden which are causing many injuries every day, which despite not being so striking, the impacts are equally as great. It is for this reason that a systematic understanding of all the factors affecting construction safety is key to implementing several management systems and technologies to help reduce accident rates. The 100 factors, descriptions and categories shown in this study provide a broad and in-depth framework that construction safety stakeholders can use to verify the measures adopted to cover the full spectrum of related elements according to each of the different areas of interest. 

As a kind of summary, this section provides some representative use cases of these factors, depending on the profile of the participants which can either affect or be affected by them, that is, the different stakeholders who can use this information:

- From the point of view of **safety managers** and **prevention technicians**, who are responsible for the design, implementation and monitoring of safety plans at construction sites: How do they organise their roadmap of prevention measures to be implemented at the construction site? What are the activities and training methods to be used? What aspects should they focus on their safety designs? What elements should they check according to the different characteristics of the construction sites? What are the various components to be verified according to the different aspects of the construction sites?
- Although safety standards and prevention measure manuals guide these practices, this study provides a detailed breakdown of the factors that safety professionals should pay attention to and the respective classifications according to the aspect of interest in which a prevention measure will be implemented.
- For example, when performing the safety review guideline, the review should be organised in terms of general aspects, materials and equipment, construction site and human aspects—worker and work team, defining specific sections and factors, which appeal to different areas and different professionals working at the construction site.
- From the point of view of **legislation**, a broad knowledge of the factors and categories allows for a better evaluation of the areas and causes of the accidents that are occurring in the sector to generate instruments to cover those gaps that have been detected and which are relevant factors and which are not yet legally covered. In this regard, it is also important to mention that the first study describing fSCPs was only found in 1998, and it was not until 2015 that authors started publishing again a significant number of publications with some factors and categories. According to this analysis of the research team, this situation seems to be peculiar, as the emergence of prevention regulations in construction safety is from the late twentieth century and very early twenty-first century. In the case of Spain, for example, construction safety regulation dates back to 1997. However, the purpose of the laws is to regulate the use of basic and general protection measures, protect the integrity of workers and provide them with legal support in the case of accidents and not necessarily to research, develop and address the problem in much more depth. This study seeks to address safety in construction more deeply, allowing multivariate analysis to go beyond mere regulatory compliance.
- From the point of view of **technology providers** and developers of training elements in construction safety, the exhaustive knowledge of the fSCPs can facilitate them to explore all the aspects to which their developments should be addressed, according to the established objectives. That is, all factors can be systematically covered and prioritised according to the areas of impact sought. For example, for developers of training experiences in mixed reality, knowledge of the factors and classifications could allow them to focus their efforts on the incorporation of specific elements that consider factors according to the objectives of the developments, putting technology at the service of specific aspects that should be considered when training workers in construction safety. On the other hand, for safety monitoring technologies in the construction site through sensors or image recognition, knowing the fSCPs allows more deeply the aspects that must be monitored, thus, improving the algorithms for the appearance of alerts and control of the interactions in the worksite.
- For many other construction professionals, such as designers or general managers, fSCPs provide in-depth knowledge of aspects that can be considered by the new methodologies incorporated in the sector to manage safety. Particularly, building information modelling (BIM) functionalities for integrating management plans and collective protection elements in digital models can be related to fSCPs to evaluate and plan the different aspects and scenarios extensively to be implemented at the construction site. On the other hand, the continuous improvement principles of the lean construction philosophy, used for the implementation of the safety and health plans at the worksite, can be aligned with the factors that influence safety, thus promoting the reduction of waste in the management and monitoring processes, driving the collaboration between policies, planning, operation and review of the plans according to the aspects and classifications delivered in this study. Furthermore, aligned with these two methodologies, the in-depth knowledge of the fSCPs is relevant for the massification of the design for prevention (PtD), to promote the incorporation of safety aspects in the early stages of the project, incorporating criteria, policies and prevention measures in the conception of the projects to reduce unsafe scenarios at the construction site.

### 7.2. Limitations

While the results and conclusions of this research are based on a systematic literature review and aligned with previous research and models, some limitations of the work need to be indicated:

- While the study did not limit the search to a specific range of years and despite the relevant number of articles selected (100) including the years 1999–2004, 2006, 2007 and 2010, no relevant papers were found that contributed to the objectives of this research (according to the criteria defined in Section 2).
- The identified factors, along with the categories and classifications shown, were established by the authors based on a systematic literature review to compile and merge several previous classifications in a more general one. Given this, certain biases in the generation of these classifications should be expected. However, the thoroughness of the methodology employed for the debugging, selection and analysis of the research incorporated and the approaches shown are considered sufficient to ensure that potential author bias was negligible and did not significantly influence the focus and objectives of the research.
- The classifications and interactions of the factors shown in Figure 11 were established according to the Construction Accident Causation Framework. This shows an overall interaction of the factor categories and not specific causalities between them. Nevertheless, establishing these relationships and quantitative study of them is interesting (but complex) for understanding these interactions in more detail given the occurrence of an accident.

## 8. Conclusions

Despite the growing incorporation of new methodologies and technologies in the construction industry, particularly a growing digitalisation with much more precise and real-time monitoring of actions, more sophisticated heavy and sensorised machinery and computer modelling of every task aimed at improving sector productivity, accident rates have not fallen, and safety management has not responding efficiently to this historical problem. Therefore, a detailed understanding of the problem is key to improving safety management and reducing risks and accidents in construction. To this end, identifying the factors that influence safety is the first step to building better potential new solutions for the current framework and, more importantly, for future scenarios of the construction sector. 

Several authors have already identified various factors that influence safety in construction [7,26,27,29,30,37] related to aspects of organisational management, worker responsibilities and capabilities, job site characteristics and equipment conditions. This background is the basis for the proposal of a classification with the interaction between these factors and groups depending on the different recognisable aspects and dimensions of the projects. This study collects these experiences and focuses on the broad identification of factors affecting construction safety, defining each of them to provide a list of elements and a description of each of them. In addition, categories of different types are established, providing a clear vision for stakeholders of the aspects of the project that correspond to each of the factors and grouping them according to the level of influence towards the potential generation of an accident.

It was found that the factors belonging to the categories of “Human aspects—worker and work team” together with “Construction Site” were those with the most significant presence in the studies reviewed. In addition, within the “General aspects” category, the factors within the sub-categories of “Culture and Climate” (50%), “Safety programmes and management systems” (44%) and “Rules and Regulations” (38%) were the most prevalent, well above the other four sub-categories of “Lesson learned from accidents” (15%), “Financial aspects and productivity” (9%), “Safety investment and cost” (9%) and “Organisation” (7%). The preponderance of “Culture and Climate” seems to be logical, since they are predominant elements in organisations that promote safety. In addition, the establishment of safety programmes is key to safety management, along with the application of associated regulations. The category of “Materials and equipment” (25%) appears in no fewer number of studies; however, a greater preponderance of “Construction Site” (39%) and “Human aspects—worker and work team” (41%) were found. In this last category, the sub-category “Communication” (77%) had the highest percentage of presence of all factors, followed by “Competency” (51%) and “Worker actions/behaviour” which was slightly lower (50%). This is followed by “Motivation” (22%) and “Work pressure” (17%). On the other hand, from the perspective of the Construction Accident Causation Framework, immediate factors were the most present in the studies reviewed (54%), followed by “Shaping factors” (31%) and “Original influences” (26%).

The results shown are of interest to safety managers to correctly identify and understand all the factors that influence safety, understand in which aspects of the project they are found and understand how they contribute to the potential generation of accidents. In general terms, all construction safety stakeholders are encouraged to propose roadmaps for (a) the development of safety and health plans, (b) construction safety regulations, (c) new technological developments and (d) methodologies for safety management, according to this proposal of factors and categories. Thus, this study sought to be a comprehensive guide to the aspects to be considered in the management and design of safety, aiming to overcome the gaps in the sector in terms of training, awareness and safety monitoring, which have led to high accident rates that have not been successfully reduced.

In addition, according to the limitations described in Section 5, it is considered interesting as a future line of research to perform quantitative and statistical analysis of the factors shown and to be able to establish relationships and weighting of the interactions of these in the route to reduce risks and avoid accidents.

## Figures and Tables

**Table 1:** Key articles’ title, year, source, field, study region and the description.

Article Title	Year	Source Title	Field	Description
Developing Key Safety Management Factors for Construction Projects in China: A Resilience Perspective [31]	2020	*International Journal of Environmental Research and Public Health*	Construction safety	The construction safety factor based on resilience theory. Thirty factors were identified and classified into four categories through a literature review. A survey was used to validate the factors. Focused on projects in China.
Construction Safety Success Factors: A Taiwanese Case Study [35]	2020	*Sustainability*	Construction safety	Thirty-three success factors of site safety management of building construction projects were classified into four categories using principal component analysis (PCA) to extract the success factors (SFs) of SSM in Taiwan. Focused on construction projects in Taiwan.
Critical Safety Factors Influencing on the Safety Performance of Construction Projects in Mongolia [15]	2020	*Journal of Asian Architecture and Building Engineering*	Construction safety	Critical safety factors influencing the safety performance of construction projects. Fifty-eight factors were identified and classified into 13 categories through a literature review. A survey was used to validate, and the factors were ranked. Focused on construction projects in Mongolia.
Analysing the Underlying Factors Affecting Safety Performance in Building Construction [10]	2020	*Production Planning & Control*	Construction safety	Factors affecting safety performance in building construction. Twenty-seven factors were identified through a literature review. A survey was used to validate the factors. Focused on construction projects in the Malaysian industry.
A Comparative Analysis of Safety Management and Safety performance in Twelve Construction Projects [33]	2019	*Journal of Safety Research*	Construction safety	Identification and analyses of 16 safety management factors influencing the safety performance of construction projects based on 20 construction projects in Norway.
Assessment of Underreporting Factors on Construction Safety Incidents in US Construction Projects [5]	2019	*International Journal of Construction Management*	Construction safety	Seven general internal and four general external factors together with 28 specific factors that influence the underreporting of construction safety incidents. Questionnaires were used to validate the factors. Focused on the US construction industry.
Construction Worker Risk-Taking Behavior Model with Individual and Organisational Factors [36]	2019	*International Journal of Environmental Research and Public Health*	Construction risks	Identification and analyses of 22 risk-taking behaviour of construction workers, associated with six types of factors, through a literature review. Questionnaires were used to validate the factors. Focused on Hong Kong industry.
Causal Factors and Connections in Construction Accidents [7]	2019	*Safety Science*	Construction accidents	Causal factors and connections in construction accidents via the analysis of 76 relatively severe construction accidents in Norway. Twenty-three factors were identified and classified into four categories.
Developing Resilient Safety Culture for Construction Projects [30]	2019	*Journal of Construction Engineering and Management*	Resilient safety culture in construction	Identification of 91 factors (in four categories) that drive resilient safety culture in the construction environment. Surveys were conducted on construction projects in Vietnam.
Factors Influencing Safety Performance on Construction Projects: A Review [37]	2018	*Safety Science*	Construction safety	Ninety factors (classified into 13 categories) influencing safety performance on construction projects through a literature review. An approach to the interaction of these factors at different levels of a construction project is shown. The proposed framework was developed based on participants whose experience was limited to projects in Iran.
Identifying and Assessing the Critical Factors for Effective Implementation of Safety Programs in Construction Projects [27]	2018	*Safety Science*	Construction safety programmes	Identification and evaluation of the causal relationships of safety programme factors in construction projects in Malaysia. Fourteen factors were identified, and five safety plan factors were identified as critical.
A Qualitative Investigation of Factors Influencing Unsafe Work Behaviors on Construction Projects [29]	2018	*WORK—A Journal of Prevention Assessment & Rehabilitation*	Unsafe work behaviours	Identification of 14 themes within four categories of factors influencing unsafe work behaviours on construction projects through field observations, in-depth interviews and focus group discussions. Focused on Iranian construction projects
Critical Success Factors for Safety Management of High-Rise Building Construction Projects in China [28]	2018	*Advances in Civil Engineering*	Construction safety	Identification and analyses of 21 critical success factors for safety management of high-rise building construction projects through semi-structured interviews and a questionnaire in China.
Factors Causing Health and Safety Hazards in Construction Projects in Pakistan [34]	2017	*Mehran University Research Journal of Engineering and Technology*	Construction safety	Identification of 23 factors influencing the safety performance of construction projects based on a literature review. A questionnaire was used to analyse and rank the factors. Focused on construction projects in Pakistan
Fuzzy Probabilistic Expert System for Occupational Hazard Assessment in Construction [42]	2017	*Safety Science*	Occupational hazard in construction	Evaluation of accidents and occupational hazards in construction based on fuzzy probabilistic rules. Forty factors (classified into six categories) influencing hazard assessment in construction were identified. The working data were obtained based on three specific construction projects in Iran.
Factors Influencing Unsafe Behaviors and Accidents on Construction Sites: A Review [20]	2014	*International Journal of Occupational Safety and Ergonomics*	Unsafe behaviours and accidents	Identification of 50 factors (classified into eight categories) influencing unsafe behaviours and accidents on construction sites through a literature review.
Critical Success Factors Influencing Safety Program Performance in Thai Construction Projects [26]	2008	*Safety Science*	Construction safety programmes	Identification and evaluation of critical success factors influencing safety programme performance in Thai construction projects. Sixteen critical factors were identified, and five safety plan factors were identified as critical. A questionnaire was used to identify and analyse the factors.

## Data Availability

Not applicable.

## Supplementary Materials

The following are available online at www.mdpi.com/article/10.3390/ijerph182010884/s1, As Supplementary Materials, you will find a table showing the intersection between the 100 factors studied and the 100 papers collected.
